# Supplementary material for: Implementation of antimicrobial stewardship programs: A study of prescribers’ perspective of facilitators and barriers
Source: PLoS One. 2024 Jan 19;19(1):e0297472. doi: 10.1371/journal.pone.0297472 (PMC10798493; doi:10.1371/journal.pone.0297472)
Supplement: S1 Questionnaire — (PDF) [file pone.0297472.s002.pdf]

QUESTIONNAIRE

SECTION A: Socio demographic characteristics of respondents

1. Hospital name ………………………………
2. Address/ location ……………………………………………………………………
3. LGA……………….. State……………………
4. What is the estimated bed capacity in your facility? …………………………………
5. Type of health care center: Primary health care ☐ General hospital ☐
   Tertiary/ Referral center ☐ Private/faith-based ☐
   Others Specify ……………………………………………………
6. Age……………………………………………
7. Gender: Female [ ] Male [ ]
8. What is your profession e.g. Medical doctor ☐ Pharmacist ☐
9. What is your designation e.g. Paediatrician, Clinical pharmacist? ……………………
10. What department/specialisation are you under e.g. internal medicine?...........................
11. Years of practice? ………………………………………..

SECTION B:

Knowledge, Attitudes, and Perceptions

1. Antibiotic resistance is a problem for my practice.
   [ ] Strongly agree
   [ ] Agree
   [ ] Neither agree nor disagree
   [ ] Disagree
   [ ] Strongly disagree

2. Antibiotic resistance is a problem in Nigeria.
   [ ] Strongly agree
   [ ] Agree
   [ ] Neither agree nor disagree
   [ ] Disagree
   [ ] Strongly disagree
3. I have seen an increase in antibiotic resistant infections among my patients over the past 5 years.
   [ ] Strongly agree
   [ ] Agree
   [ ] Neither agree nor disagree
   [ ] Disagree
   [ ] Strongly disagree

4. Inappropriate antibiotic **prescribing** is a problem only in outpatient healthcare settings.
   [ ] Strongly agree
   [ ] Agree
   [ ] Neither agree nor disagree

[ ] Disagree
[ ] Strongly disagree

5. Inappropriate antibiotic prescribing in outpatient healthcare settings accelerates the emergence of antibiotic-resistant bacteria.
   [ ] Strongly agree
   [ ] Agree
   [ ] Neither agree nor disagree
   [ ] Disagree
   [ ] Strongly disagree

6. Inappropriate antibiotic **prescribing** is a problem in my practice.
   [ ] Strongly agree
   [ ] Agree
   [ ] Neither agree nor disagree
   [ ] Disagree
   [ ] Strongly disagree

7. Inappropriate antibiotic **dispensing** is a problem in my practice.
   [ ] Strongly agree
   [ ] Agree
   [ ] Neither agree nor disagree
   [ ] Disagree
   [ ] Strongly disagree

8. Overall, how much pressure do you experience from patients to prescribe antibiotics?
   [ ] Very high pressure
   [ ] High pressure
   [ ] Moderate pressure
   [ ] Low pressure
   [ ] No pressure at all

SECTION C
Acceptability of Stewardship Approaches/Interventions

1. Does your facility have a formal antimicrobial stewardship program accountable for ensuring appropriate antimicrobial use? Yes [ ] No [ ] I don't know [ ]

2. Is an antimicrobial stewardship team available at your facility (e.g., greater than one staff member supporting clinical decisions to ensure appropriate antimicrobial use)?
   Yes [ ] No [ ] I don't know [ ]

3. Are you a member of your facility antimicrobial stewardship team? Yes [ ] No [ ]

4. What level of authority do you have in implementing antibiotic stewardship interventions in your healthcare facility?

    a. I am the primary decision maker for my practice.
    b. I can provide input, but the final decision would be made by practice leadership.
    c. I do not have the authority within my practice to make decisions related to quality improvement interventions.

5. Please let us know to what extent you agree or disagree with each of the following statements about antibiotic stewardship.

    Use a scale of 1 to 5 where

1= Strongly disagree
2 = Disagree
3 = Neither agree nor disagree
4 = Agree
5 = Strongly agree

    a. Antibiotic stewardship efforts implemented by providers will be ineffective unless also paired with efforts aimed at educating patients about antibiotic resistance and use. [ ]
    b. Tracking the appropriate use of antibiotics would be difficult to do in an accurate and fair manner. [ ]
    c. Antibiotic stewardship programs are needed in healthcare settings to effectively deal with antibiotic resistance. [ ]
    d. I prescribe antibiotics appropriately based on laboratory diagnosis. [ ]
    e. I prescribe antibiotics appropriately based on clinical manifestations [ ]
    f. I would need a lot of help to implement antibiotic stewardship interventions in my healthcare facility. [ ]
    g. I feel a reasonable discussion with my patients about the value of an antibiotic for their current symptoms is about all I need to do to support antibiotic stewardship efforts. [ ]
    h. Practice-based reporting requirements for antibiotic use would be too burdensome. [ ]
    i. Health plans are in a good position to give feedback on antibiotic use to medical practices. [ ]
