# Supplementary material for: Implementation of antimicrobial stewardship programs: A study of prescribers’ perspective of facilitators and barriers
Source: PLoS One. 2024 Jan 19;19(1):e0297472. doi: 10.1371/journal.pone.0297472 (PMC10798493; doi:10.1371/journal.pone.0297472)
Supplement: S1 File — (PDF) [file pone.0297472.s003.pdf]

Interview Guide

Facilitators and Barriers to implementation of Antimicrobial Stewardship: A Study of Prescribers perspective using mixed method

Introduction

Thank you for talking with me today. I am interested in learning more about your thoughts, perception and experiences in implementing Antimicrobial Stewardship. I am going to ask you some questions about these topics. (Please remind the client that the interview will be audio-recorded and pseudo names or nicknames will be used during the discussion).

Please know that there is no right or wrong answer to any of the questions that I will ask and the discussion will be driven by your responses – my only goal is to facilitate to get more clarity on the statements you share. Occasionally, I will take notes to remind me to ask follow-up question if needed. You are under no obligation to talk about anything that you are not comfortable discussing with me. This session is being recorded, as noted in the consent form you signed before so that none of your informative comments and feedback will be missed. Please let me know if you have any questions or concerns before we begin.

Study ID……………………………

Section A: Basic Information/Background Characteristics

1. Age……
2. Gender (1) Female (2) Male
3. Profession: (1) Physician (2) Pharmacist
   (3) Others please specify ……………………………………
4. Healthcare Facility: (1) Private facility (2) General Hospital (3) Private Pharmacy (4) General pharmacy (5) Others……………………………..

Section B: Implementation of Antimicrobial Stewardship (AMS)

5. Is your leadership concerned about the use of antimicrobials?
   a) Probe: If so, in what way
6. Is there anything about the way your hospital is organized that could facilitate the prescribing of antimicrobials? With organization, I mean schedules, time, staff, medical records, charts and so on.
7. What are your thoughts on antimicrobial resistance?

8. How is your access to information and help on antimicrobial prescribing when you need it?
9. How is the process of retrieving microbiological test results?
10. Do you have a stewardship program in your facility?
    a) Probe: How long has this program been in operation in your facility?
    b) Probe: Do you play a role in the stewardship program?

Describe your work/role in relation to AMS or as a champion?
    a) Probe: How have you used your role/position to influence AMS?
11. Tell me about the factors that you think will promote the implementation of rationale antibiotic prescribing?
    a) Probe: What are the things that help with proper implementation?
12. How will you describe the factors that inhibit the implementation of best practices?
    a) Probe: What are the concerns with regards proper implementation?
    b) Probe: What are some of the things that make it difficult to implement AMS?
13. Do you think that there are changes in the existing practices?
    a) Probe: What are these changes you have encountered/witnessed?
14. What is your perceived impact of best practices with regards to AMS?
    a) Probe: On a scale of 1-10, how will you rate AMS in your facility?
15. What strategies are adopted by your facility in implementing AMS
    a) Probe: What are the changes you have encountered/witnessed?
16. What strategies do you think will facilitate AMS?
17. Do you have suggestions or recommendations in relation to AMS in Nigeria?
18. What measures do you think would be most useful in order to improve antimicrobial prescribing?
19. Is there anything else you would like to add before we finish?

Thank you for participating
