# Supplementary material for: Implementation of antimicrobial stewardship programs: A study of prescribers’ perspective of facilitators and barriers
Source: PLoS One. 2024 Jan 19;19(1):e0297472. doi: 10.1371/journal.pone.0297472 (PMC10798493; doi:10.1371/journal.pone.0297472)
Supplement: Attachment — Submitted filename: Comments for review AMS paper.docx [file pone.0297472.s005.docx]

**Abstract**…… programs and interventions in one sentence side-by-side

…..creating awareness and training and inadequate personnel respectively “not clear”. OBSERVE PUNCTUATION MARKS. Which are the facilitators, and which are the barriers. That needs to clearly come out in your abstract.

……”resource-poor” and not resource poor

**Introduction**

….”emergence and transmission of AMR in Nigeria” change to “**transmission of AMR genes among disease pathogens**”. Antimicrobial resistance doesn’t seem to be transmitted. It’s the genes that are the problem.

**Change** ….”low-and-middle” **to** ….”low- and middle-income….” That isn’t one word

Methods….

…..”antibiotic- related” change to **antibiotic-related**

….”faith- based” change to **faith-based**

**What ethical issues could be anticipated from this study and how were these issues addressed?**

**How many FGDs were conducted and do the authors think the number of FGDs were sufficient?**

**How about conducting In-depth interviews with these stakeholders? Could that had given better perspective to this study?**

**What software was used for the qualitative coding and analysis?**

**……”**SPSS version 26.0For…..” Change to **………….SPSS version 26.0. For**………….

**Results**

**……”**About one-half”……..Correct to **“About half”** Is there anything like two-half???

68% is about two-third and not about one-half, kindly correct!!!

What the authors described as facilitators are suggested approaches to improve AMS rather than facilitators!! Correct the title to reflect appropriately what is reflected by the results!!

The entire barrier section has not supporting quotes! Why??

What is the difference between the recommended strategies and facilitators as captured in the study??
