# Supplementary material for: Implementation of antimicrobial stewardship programs: A study of prescribers’ perspective of facilitators and barriers
Source: PLoS One. 2024 Jan 19;19(1):e0297472. doi: 10.1371/journal.pone.0297472 (PMC10798493; doi:10.1371/journal.pone.0297472)
Supplement: Attachment — Submitted filename: Response to reviewers.docx [file pone.0297472.s006.docx]

Journal Requirements:

When submitting your revision, we need you to address these additional requirements.

| S/N | Editors’ comments | Authors response |
| --- | --- | --- |
| 1 | Please ensure that your manuscript meets PLOS ONE's style requirements, including those for file naming. | The manuscript has been revised to conform to the journals style |
| 2 | Please provide additional details regarding participant consent. In the ethics statement in the Methods and online submission information, please ensure that you have specified (1) whether consent was informed and (2) what type you obtained (for instance, written or verbal, and if verbal, how it was documented and witnessed). If your study included minors, state whether you obtained consent from parents or guardians. If the need for consent was waived by the ethics committee, please include this information.  If you are reporting a retrospective study of medical records or archived samples, please ensure that you have discussed whether all data were fully anonymized before you accessed them and/or whether the IRB or ethics committee waived the requirement for informed consent. If patients provided informed written consent to have data from their medical records used in research, please include this information. | “Participants were provided detailed written consent forms about the study and informed of their rights to participate or decline in the study. Participants, who agreed to participate, signed a written consent form, and participated in the study. Information obtained from participants were treated as confidential and with no identifier.”  This is clearly stated in the ethical statement session and has now been placed appropriately in the methods section.  The study did not utilize patients’ sample or report patients’ data |
| 3 | Your ethics statement should only appear in the Methods section of your manuscript. If your ethics statement is written in any section besides the Methods, please move it to the Methods section and delete it from any other section. Please ensure that your ethics statement is included in your manuscript, as the ethics statement entered into the online submission form will not be published alongside your manuscript. | The ethics statement has been moved from the initial position to the methods section. Thanks |
| 4 | Please upload a copy of Supporting Information Figure/Table/etc. Supplementary material (Suppl 1&2) which you refer to in your text on page 4. | All figures and tables from this study has been included in the main submission.  Supplementary materials 1 (Study questionnaire) and 2 (interview guide) was uploaded with the manuscript and has now been properly labeled as S1 and S2 |

**Reviewer #1**

| **S/N** | **Reviewer’s comments** | **Authors response** |
| --- | --- | --- |
| 1 | General comment: This topic is key in the fight against AMR and the authors are commended for the efforts. However, I strongly suggest that the authors read through the entire manuscript for corrections of some grammatical errors. | Thank you for taking time out to review our manuscript.  The authors have read through the manuscript and corrected grammatical errors |
| 2 | **Abstract** a. In the methods, I suggest you use “focus group discussion” instead of “focused group discussion”.  b. In the methods, no clear details on the analysis of the quantitative data.    c. In the results, you may write “thirty” or “30” instead of “thirty (30)”.  d. Regarding the means, having stated the mean and standard deviation, there may be no need to still state the range. Range is used as the measure of dispersion for skewed data while standard deviation is used as the measure of dispersion for data with normal distribution.  e. The use of AMS in the results was not defined. The authors may have to write it out in full as was done in the conclusion.  f. Having defined ASPs, there should be consistency in its use rather than writing in full as seen in the last sentence of the conclusion. | Done!  A statement of the quantitative data analysis approach used has been added.  Corrected  This has been corrected as mean and standard deviation.  This has now been written in full at its first appearance.  Done! |
| 3 | 3. Introduction a. Define AMR in its first use before continuing with the abbreviation.  b. Having defined ASPs, there should be consistency in its use rather than writing in full again.  c. In page 3, LMICs should be stated in the first paragraph before its continuous use. | Done  Done  Done |
| 4 | 4. Methods a. In the methods, I suggest you use “focus group discussion” instead of “focused group discussion”.  b. Under the “Study Design and Sampling Technique”, some details on the analysis of the qualitative data should be moved to the data analysis subsection of the methods.  c. The authors stated in the results section that 20 participants were interviewed for the qualitative component of the study. Were these key informant interviews or FGDs? If they were FGDs, how many FGDs were conducted and what was the number of participants per FGD? Or  were all 20 participants included in one FGD? | Corrected  The analysis details have been moved to the data analysis section.  20 participants volunteered and participated in the focus group discussions. Two FDGs were conducted comprising of 10 participants each. This has been clarified |
| 5 | 5. Results a. Take note of 2c and 2d above.  b. The results should be reported in past tense. See example from the results section - “About one-half of the participants either do not have or are not sure if there exists an antimicrobial stewardship team in their facility while 28% were members of their facility stewardship team” | Done  Done |
| 6 | 6. Discussion a. The authors made a good attempt to discuss their results. | Thank you |
| 7 | 7. References a. About half of the references are more than 5 years old. I recommend that the authors add more recent references (within the last 5 years). | Some more recent references have been added |

**Reviewer #2**: This work is so important, particularly as Nigeria is implementing NAP. The study will make a great contribution. However, there are issues that need to be considered and are highlighted below. There are valuable questions about the methods and results that need to be addressed to give these readers that insight that is required for this valuable study.

| **S/N** | **Reviewer’s comments** | **Authors response** |
| --- | --- | --- |
| 1 | Abstract…… programs and interventions in one sentence side-by-side  …..creating awareness and training and inadequate personnel respectively “not clear”. OBSERVE PUNCTUATION MARKS. Which are the facilitators, and which are the barriers. That needs to clearly come out in your abstract.   ……”resource-poor” and not resource poor | Done.  This has been rephrased for clarity.  Done |
| 2 | Introduction ….”emergence and transmission of AMR in Nigeria” change to “transmission of AMR genes among disease pathogens”. Antimicrobial resistance doesn’t seem to be transmitted. It’s the genes that are the problem.  Change ….”low-and-middle” to ….”low- and middle-income….” That isn’t one word | Done  Done |
| 3 | Methods…. …..”antibiotic- related” change to antibiotic-related  ….”faith- based” change to faith-based  What ethical issues could be anticipated from this study and how were these issues addressed?    How many FGDs were conducted and do the authors think the number of FGDs were sufficient?    How about conducting In-depth interviews with these stakeholders? Could that had given better perspective to this study?    What software was used for the qualitative coding and analysis?    ……”SPSS version 26.0For…..” Change to ………….SPSS version 26.0. For…………. | Done  Done  Ethical clearance for human subject participation to conduct this study was obtained from the NIMR Institutional Review Board. However, participants were informed of their right to either participate or decline to participate in the study and only participants who gave their informed consent by signing the written consent form were included in the study.  Two sets of FGDs were conducted with 10 participants in each set. One set was conducted with medical doctors and another set was conducted with pharmacists. Participation in the FGDs was voluntary. The FGDs had proper representation of participants based on demographics (Gender, age,) type of practice (public, private), range of experiences (early career, mid-career and advanced career clinicians and pharmacists) and the numbers of specialist in each group (10) providing unique and vast knowledge. The FGDs ended when the participants did not provide new or additional data as the same thematic areas were being re-emphasized and re-explained.  This information has been included in the methods section (Focus Group Discussion)  FGDs and In-depth interviews both offer unique advantages for qualitative research. However, for the purpose of this study, we aimed to gather individual stakeholders for a collaborative discussion on issues around implementing ASPs rather than individual perspective.  In addition, participants were vocal, and understood the thematic focus and provided comprehensive discussions, rich understanding of the study context.  Data analysis was done manually by a team of qualitative research experts and the processes has been explained in the data analysis section.  Done |
| 4 | Results ……”About one-half”……..Correct to “About half” Is there anything like two-half??? 68% is about two-third and not about one-half, kindly correct!!!  What the authors described as facilitators are suggested approaches to improve AMS rather than facilitators!! Correct the title to reflect appropriately what is reflected by the results!!    The entire barrier section has not supporting quotes! Why??  What is the difference between the recommended strategies and facilitators as captured in the study?? | Done  Thank you, the participants responded based on the question asked about factors that could facilitate antimicrobial stewardship. The response documented were the first-hand accounts of what they believed were the factors that will motivate prescribers and healthcare facilities to engage in antimicrobial stewardship. However, we recognize that some participant-identified facilitators also featured/doubled as recommendations hence the implicated themes have now been moved to and discussed under recommendations.  Supporting quotes have been added to this section.  The respondents provided insights on the facilitators of AMS which are factors that the respondents believed would favour, motivate, or help individual prescribers as well as healthcare facilities to engage in a rational prescription of antibiotics while also recommending approaches for effective ASP implementation. |
