# Supplementary material for: Implementation of antimicrobial stewardship programs: A study of prescribers’ perspective of facilitators and barriers
Source: PLoS One. 2024 Jan 19;19(1):e0297472. doi: 10.1371/journal.pone.0297472 (PMC10798493; doi:10.1371/journal.pone.0297472)
Supplement: Attachment — Submitted filename: Response to reviewers comments_2nd revision.docx [file pone.0297472.s007.docx]

Response to Reviewers comments

| S/N | Editorial comments | Author’s response |
| --- | --- | --- |
| 1. | Please provide additional quotes for facilitators of AMS. For each of the points raised, kindly provide at least two quotes from the FGD. Only one point "Having specialized and dedicated Teams" was supported by one quote. | Additional quotes have been provided for the facilitators with at least two quotes per listed facilitator |
|  | Also provide at least two quotes for each barrier to the uptake of stewardship programs as provided by the respondents during the FGD.  As it currently stands each point has one supporting quote. Kindly provide one additional quote to the one already provided for clarity and consistency please. | One additional quote has been provided for the barriers with at least two quotes per listed barrier |
|  | Once an acronym/abbreviation has been defined upon it's first use, it is expected that you use the acronym in the rest of the manuscript. For instance AMR has been defined previously so kindly correct page 22 line 3 "lack of data on antimicrobial resistance" to "lack of data on AMR". Please fix this issue wherever it occurs in the manuscript. Also check for consistency with other abbreviations used. This applies to AMS and antimicrobial stewardship. So kindly fix this as well all through the manuscript. | Done!  The defined terms have been replaced with acronyms all through the manuscript. |
|  | Reviewer #2: . | Thank you for your review comments |
|  | I still don't understand the reason for leaving out the quotes for some of the facilitation | Quotes have been provided for all the facilitators and barrier initially without quotes and additional quotes added to the existing with a target of two quotes per listed factor |
|  | The author(s) did not adequately address the issue of barriers. I have stated that the barriers identified by the author(s) did not align with the quotes and read more like suggestions to improvement. | The authors presented respondents identified barriers and perceived challenges to the effective implementation of ASP in their various facilities which was summarized under 5 themes. We have added additional quotes to provide more context to their stated barriers.  We also want to mention that majority of the facilities (except one facility) did not have a standard antimicrobial stewardship program, rather had stewardship teams operating at various levels and the respondents discussed their perceived facilitators and barriers to implementation of a stewardship program in their facilities. Most of the responses were context specific for a resource poor setting. This has been stated in the limitation section |
|  | Again, I am wondering if the framework was used to develop a discussion guide because if you begin to ask questions until respondents began to repeat their responses then I wonder of there wad and FGD guide and if it was followed. | The FDG was conducted using a semi-structured interview guide which is provided as a supplementary material (S2) with probes to allow flexibility for the respondents provide in-depth insight on emerging themes and sub-themes and also exploring new ideas with follow-up questions until no new information is being provided |
|  | Why did the authors decide to leave recommendations in table and the others in full writing? | The Use of tables for the recommendations allowed for a more detailed presentation of the identified themes and sub-themes and their supporting quotes  However, the implication of all identified facilitators and barriers as well as recommendations were further discussed in context and in comparison, with the quantitative data in the discussion session |
